# Resection of Motor Eloquent Metastases Aided by Preoperative nTMS-Based Motor Maps—Comparison of Two Observational Cohorts

**DOI:** 10.3389/fonc.2016.00261

**Published:** 2016-12-21

**Authors:** Sandro M. Krieg, Thomas Picht, Nico Sollmann, Ina Bährend, Florian Ringel, Srikantan S. Nagarajan, Bernhard Meyer, Phiroz E. Tarapore

**Affiliations:** ^1^Department of Neurosurgery, Klinikum rechts der Isar, Technische Universität München, Munich, Germany; ^2^Department of Neurosurgery, Charité – Universitätsmedizin Berlin, Berlin, Germany; ^3^Biomagnetic Imaging Laboratory, Department of Radiology, University of California San Francisco, San Francisco, CA, USA; ^4^Department of Neurological Surgery, University of California San Francisco, San Francisco, CA, USA

**Keywords:** brain metastases, matched pair, preoperative mapping, Rolandic region, transcranial magnetic stimulation

## Abstract

**Introduction:**

Preoperative mapping of motor areas with navigated transcranial magnetic stimulation (nTMS) has been shown to improve surgical outcomes for peri-Rolandic lesions and, in particular, for gliomas. However, the impact of this technique on surgical outcomes for peri-Rolandic metastatic lesions is yet unknown.

**Objective:**

To investigate the impact of nTMS on surgical outcomes for peri-Rolandic metastatic lesions, various clinical parameters were analyzed in our international study group.

**Methods:**

Two prospectively enrolled cohorts were compared by investigating patients receiving preoperative nTMS (2010–2015; 120 patients) and patients who did not receive preoperative nTMS (2006–2015; 130 patients). Tumor location, pathology, size, and preoperative deficits were comparable.

**Results:**

The nTMS group showed a lower rate of residual tumor on postoperative magnetic resonance imaging (odds ratio 0.3025; 95% confidence interval 0.1356–0.6749). On long-term follow-up, surgery-related paresis was decreased in the nTMS group (nTMS vs. non-nTMS; improved: 30.8 vs. 13.1%, unchanged: 65.8 vs. 73.8%, worse: 3.4 vs. 13.1% of patients; *p* = 0.0002). Moreover, the nTMS group received smaller craniotomies (nTMS: 16.7 ± 8.6 cm^2^ vs. non-nTMS: 25.0 ± 17.1 cm^2^; *p* < 0.0001). Surgical time differed significantly between the two groups (nTMS: 128.8 ± 49.4 min vs. non-nTMS: 158.0 ± 65.8 min; *p* = 0.0002).

**Conclusion:**

This non-randomized study suggests that preoperative motor mapping by nTMS may improve the treatment of patients undergoing surgical resection of metastases in peri-Rolandic regions. These findings suggest that further evaluation with a prospective, randomized trial may be warranted.

## Introduction

One indication for surgical resection of cerebral metastases is a focal motor deficit. In these patients, early surgery is often recommended so as to preserve existing function and, hopefully, to allow recovery of lost function. In these cases, surgeons must frequently contend with a lesion or lesions that threaten the motor cortex or subcortical motor tracts, and any technique that helps to identify and preserve those functional regions is valuable (1).

Continuous motor-evoked potential (MEP) monitoring and subcortical electrical stimulation are two well-established techniques to monitor and map functional integrity of the motor system. Another technique, navigated transcranial magnetic stimulation (nTMS), which uses magnetic pulses to activate small regions of cortex, has demonstrated the ability to map the motor system in the preoperative setting. For supratentorial lesions located in motor eloquent areas, especially gliomas, three prior studies have demonstrated improvement in outcomes when preoperative functional mapping of motor areas is also performed by nTMS (2–4).

In comparison to other preoperative mapping techniques such as functional magnetic resonance imaging (fMRI) and magnetoencephalography (MEG), nTMS correlates better with intraoperative direct cortical stimulation (DCS) (5, 6). This correlation has been validated in international studies, which have also shown nTMS to be a helpful tool for surgical planning (6, 7).

Although the positive impact of nTMS on surgical indication, approach planning, and functional outcome has been shown for gliomas, similar effects have yet to be demonstrated in the management of metastatic lesions (2–4). This multicenter study aims to compare the surgical outcome of patients with motor eloquent metastatic lesions who received preoperative nTMS-based motor mapping with those that did not. In so doing, we aim to characterize the impact of preoperative nTMS in the management of these challenging lesions.

## Materials and Methods

### Investigated Patient Cohorts

Inclusion criteria were (1) planned resection of one supratentorial metastasis and (2) anatomical association between tumor and Rolandic cortex as seen on magnetic resonance imaging (MRI). Eligibility for surgery was discussed on a case-by-case basis with each patient and reviewed by an interdisciplinary tumor board, which included a practitioner of stereotactic radiosurgery (SRS). A plan for surgical resection required the consent of all disciplines (neurosurgery, neurooncology, radiation oncology, and medical oncology) according to the present guidelines (8, 9). Surgery was recommended for patients presenting with disabling motor weakness or progressive disease despite chemo- or radiotherapy. In all centers, the surgical goal was complete resection. Primary SRS was the treatment of choice for patients with small tumors (relative cutoff value 2 cm max. diameter) who did not meet the aforementioned criteria.

Between 2010 and 2015, 120 consecutive patients with presumed motor eloquent supratentorial metastatic lesions were prospectively enrolled and underwent craniotomy in the three participating institutions. Presumed motor eloquent location was defined as a location of the lesion in or adjacent to the precentral gyrus on contrast-enhanced T1 MRI images. A control group of 130 consecutive patients who did not undergo nTMS due to organizational reasons (missing staff, maintenance, or technical problems) were identified and analyzed as well. This control group contained patients from each institution and included cases performed from 2006 to 2015 with a comparable distribution of tumor types, tumor locations, and treatment modalities. The characteristics of the nTMS and the non-nTMS group are outlined in Table 1. With the exception of tumor size, both groups were highly comparable. Only mean tumor diameter differed by 3.0 mm (Table 1). Data analysis was performed blinded to the assigned group. Mean follow-up for clinical evaluation in the outpatient departments was 9.9 ± 10.5 months (median 6.0 months, range 0.3–57.0 months) in the nTMS and 9.8 ± 10.8 months (median 5.0 months, range 0.3–55.1 months) in the non-nTMS group.

**Table 1:** **Patient data**.

		nTMS	Non-nTMS	*p*-Value
Mean age (years ± SD)		59.1 ± 11.9	62.3 ± 12.4	0.0394
Gender (%)	Male	48.3	51.5	0.6126
	Female	51.7	48.5	
Preoperative paresis (%)	None	48.3	60.8	0.1283
	Mild	33.3	26.9	
	Severe	18.4	12.3	
Location (%)	Frontal lobe	72.5	69.2	0.5701
	Parietal lobe	27.5	30.8	
Histology (%)	NSCLC	35.7	31.5	0.2146
	Breast	16.3	18.5	
	Melanoma	10.2	18.5	
	Colon	7.1	8.1	
	RCC	5.1	7.3	
	Seminoma	3.1	0.0	
	Other	22.5	16.1	
Mean tumor diameter (cm)		2.8 ± 1.1	3.1 ± 1.4	0.0610
Mean follow-up (months)		9.9 ± 10.5	9.8 ± 10.8	0.9588

*Detailed overview on age, gender, preoperative neurological status, location, histology, tumor diameter, and mean follow-up*.

*NSCLC, non-small cell lung cancer; RCC, renal cell cancer. Preoperative paresis: none, no paresis; mild, British Medical Research Council (BMRC) grade of muscle strength ≥4/5; severe, BMRC grade of muscle strength ≤3/5*.

### Ethics Approval and Consent to Participate

This study was approved by the local institutional review boards of all three centers according to ethical standards of the Declaration of Helsinki. Written informed consent was obtained prior to every nTMS examination from each patient. Ethics Committee Registration Numbers: 2793/10, 5497/12, 10-02932, and EA4/007/06.

### Perioperative MRI

All enrolled patients underwent preoperative MRI scans using 3-T MR scanners in combination with 8-channel phased array head coils (Achieva 3 T, Philips Medical Systems, The Netherlands B.V.; Siemens Magnetom 3 T, Munich, Germany; GE Excite 3 T, Fairfield, CT, USA) for contrast-enhanced 3D gradient echo sequence, FLAIR, and diffusion tensor imaging (DTI). The contrast-enhanced 3D gradient echo sequence dataset was transferred to the nTMS system (eXimia 3.2 and eXimia 4.3, Nexstim Oy, Helsinki, Finland). Depending on each institution’s standards, every patient underwent either a contrast-enhanced postoperative computed tomography (CT) or MRI scan the day after surgery to evaluate the extent of resection (EOR). MRI included T1 sequences (±contrast enhancement), diffusion-weighted imaging to detect any postoperative ischemic events, and FLAIR. Unexpected residual tumor was defined as the presence of residual tumor on postoperative MRI scans despite the surgeon’s impression being stated as gross total resection (GTR). For regular follow-up examinations, MRI scans were performed every 3 months and reviewed for recurrent metastases since neurological status during follow-up was only considered without tumor recurrence. Moreover, all performed MRI and CT scans were evaluated in regular imaging meetings including board certified neurosurgeons and neuroradiologists.

Since craniotomy was also compared between groups, craniotomy size was analyzed by postoperative MRI or CT scans in all 250 patients. Analysis was done on a case-by-case basis by a neurosurgeon; by doing so, we took account for oval-shaped as well as rectangular bone flaps. Anterior–posterior (AP) and lateral extent of craniotomy were evaluated as well.

### Clinical Assessment

Clinical assessment was performed by experienced neurosurgical staff including nurse practitioners, residents, and attending surgeons. All patients were evaluated for muscle strength, sensory function, and coordination before surgery. After surgery, neurological status was assessed again after awakening from anesthesia and each day until discharge, again at 6–8 weeks postoperatively, and during clinical follow-up visits every 3 months, depending on tumor type. New surgery-related motor deficits were differentiated as transient and permanent.

A new or worsened postoperative motor deficit which did not return to the preoperative baseline within 8 weeks from surgery was defined as a new permanent paresis. A new or worsened postoperative motor deficit that resolved within the 8-week follow-up interval was defined as a transient paresis. Every patient with a new motor deficit directly after surgery underwent a postoperative CT scan to exclude secondary hemorrhage. Muscle reflex evaluation and resection location were used to differentiate postoperative supplementary motor area deficits from primary motor area or corticospinal tract (CST) deficits.

### Motor Mapping by nTMS

The three participating institutions were equally experienced in nTMS motor mapping during the enrollment period and used the same technique throughout. In brief, the procedure is performed as follows: a magnetic coil placed on the patient’s scalp induces a transient magnetic field, which then induces a perpendicular electric field in the underlying cortex (10–13). The shape, strength, and penetration depth of the elicited electric field depend on the stimulation intensity, design of the coil, and the shape of the brain itself (12, 13). The electric field is able to modulate cortical neuronal activation for the period of stimulation (10, 14), resulting in action potentials from the neurons within the affected region (11). The action potential is transmitted via the CST to downstream muscle groups, the activations of which are measured as MEPs by electromyography (EMG) electrodes placed over the muscle belly. By integrating a stereotactic neuronavigation system with transcranial magnetic stimulation (TMS) (so-called “navigated TMS,” or nTMS), we have gained the ability to individualize the mapping procedure to a given patient’s anatomy and identify exactly the site of cortical stimulation (15). Thus, nTMS and DCS apply the same neurophysiological principles, but nTMS has the advantage of being non-invasive.

All enrolled patients of the nTMS group underwent primary motor cortex mapping the week before surgery using a widely accepted, previously published protocol (6, 16). Briefly, resting motor threshold (rMT) was identified first; subsequently, nTMS motor mapping was performed using a stimulation intensity of 110% rMT for the upper and 130% for the lower extremity (6). The nTMS mapping session started at the anatomically most lateral border of the hand knob and was then performed in 3–5 mm steps perpendicular to the nearest sulcus until nTMS did not evoke any further MEP in any direction. Each cortical point at which an MEP above 50 µV was elicited was defined as a motor-positive mapping point, and the associated muscle groups were identified by examining EMG responses. After an additional post hoc analysis of each stimulation spot, all motor-positive mapping points were then exported from the nTMS system to the intraoperative neuronavigation system using DICOM format files. All three centers used the same two nTMS systems (eXimia 3.2 and eXimia 4.3, Nexstim Oy, Helsinki, Finland), which use a biphasic figure-8 TMS coil with a 50-mm magnetic stimulator attached to an infrared tracking system (Polaris Spectra, Waterloo, ON, Canada), as previously described (6, 7, 16). All investigators underwent manufacturer certification before entering the study.

### Intraoperative Neuronavigation

**Figure 1:**
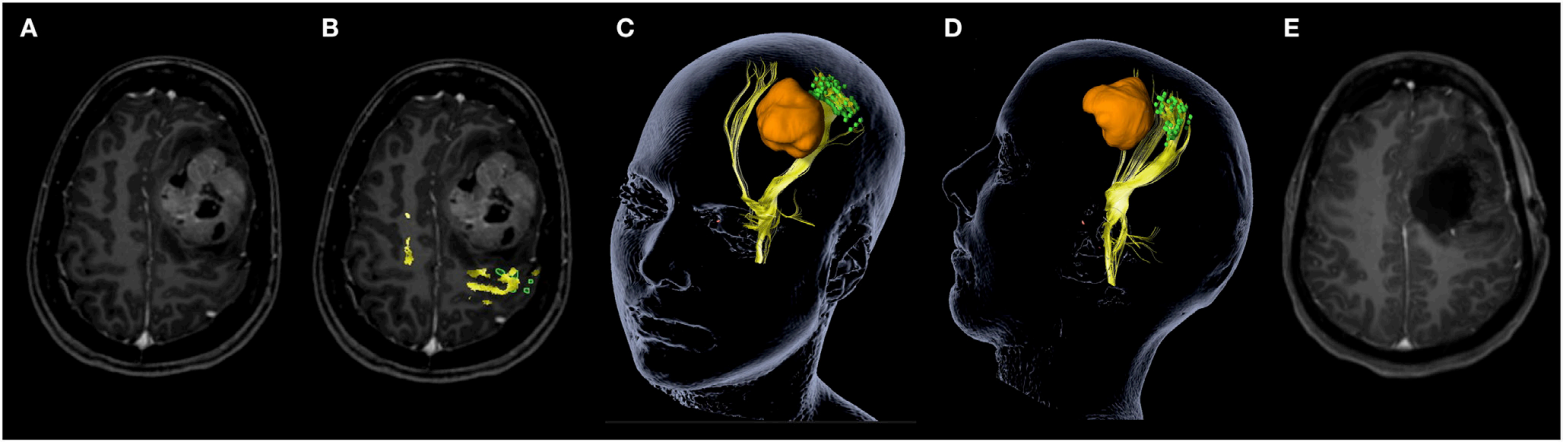
**Navigated transcranial magnetic stimulation (nTMS) data clarifies functional anatomy for patient counseling**. This screenshot demonstrates how nTMS data can clarify functional anatomy in this young patient with a large Ewing sarcoma metastasis. Tumor location is shown on contrast-enhanced magnetic resonance imaging (MRI) without **(A)** and with nTMS-positive motor areas (green) including nTMS-based diffusion tensor imaging fiber tracking of the corticospinal tract (yellow) **(B)**. The 3D reconstruction **(C,D)** shows the same coloring including visualization of the tumor in orange, which allows optimal patient counseling explaining functional anatomy in relation to the tumor. Postoperative MRI scan shows gross total resection **(E)**.

Intraoperative neuronavigation (Vector Vision 2^®^, Vector Vision Sky^®^, or Curve; BrainLAB AG, Feldkirchen, Germany) was used in every case in both the nTMS and the non-nTMS group. nTMS data were included in the neuronavigation system for the nTMS group by exporting nTMS-positive motor areas as DICOM files and importing them to the neuronavigation planning unit (BrainLAB iPlan^®^ Net Cranial 3.0.1; BrainLAB AG, Feldkirchen, Germany). nTMS-positive motor areas were fused to a 3D image set of a T1-weighted 3D gradient echo sequence and FLAIR and defined as objects by simple auto segmentation thus making them available as 3D objects for the intraoperative use, as described earlier (17). The process of implementing nTMS data into the neuronavigation planning took 2–5 min per patient. The result, including nTMS-based DTI fiber tracking (DTI FT) for visualization of the CST, is shown in Figure 1.

### Surgical Technique

Intraoperative neuromonitoring (IOM) was used at the surgeon’s discretion, depending on the proximity of the lesion to motor eloquent cortex or CST. Identification of CST was based on anatomic landmarks in the non-nTMS group. IOM included mapping and monitoring by monopolar DCS for evoking MEPs as also outlined in earlier reports (18, 19). Surgical technique and surgeons’ experience did not vary significantly between groups.

### Statistical Analysis

To test the distribution of several attributes Chi-square or Fisher Exact tests were used. Differences between nTMS and non-nTMS patients were analyzed by using the Mann–Whitney–Wilcoxon test for non-parametric rank comparisons, and the *t*-test for parametric distributions. Level of significance was 0.05 (two-sided) for each statistical test, and results are presented as mean ± SD as well as odds ratios (ORs) with 95% confidence intervals (CIs) (GraphPad Prism 6.0, La Jolla, CA, USA).

## Results

### nTMS Motor Mapping Prior to Surgery

All 120 patients of the nTMS group underwent preoperative mapping of the primary motor cortex by nTMS. No patient was unable to undergo nTMS, and no adverse events were reported.

### Influence on Surgery

#### Craniotomy Size

**Figure 2:**
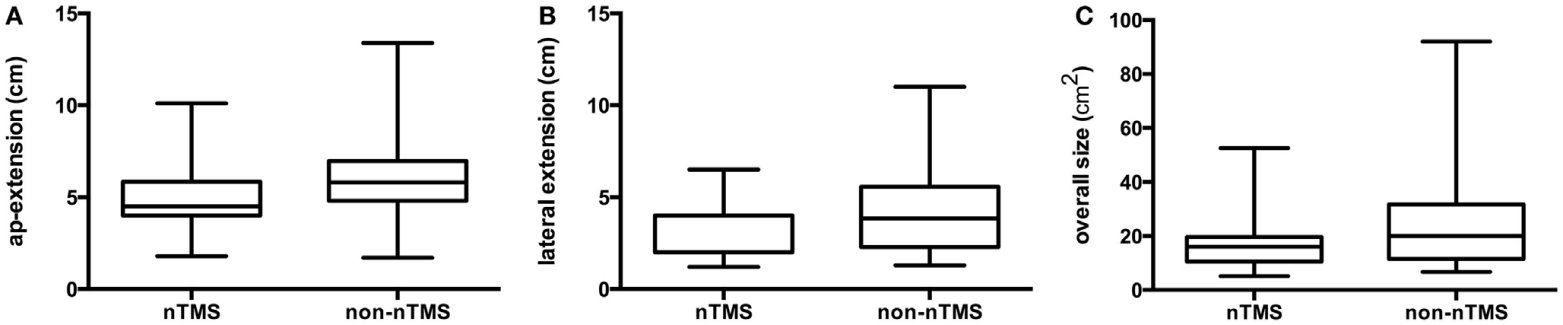
**Size of craniotomy**. This boxplot of the extent of craniotomy for navigated transcranial magnetic stimulation (nTMS) and non-nTMS patients shows with median, min- and max-whiskers, and quartile-boxes the differences between both groups for anterior–posterior (*p* < 0.0001) **(A)**; and lateral direction (*p* = 0.0166) **(B)**; as well as overall craniotomy size (*p* < 0.0001) **(C)**.

Extension of the craniotomy in the AP direction was 5.0 ± 1.6 cm for the nTMS (median 4.5 cm, range 1.8–10.1 cm) and 6.1 ± 2.1 cm (median 5.8 cm, range 1.7–13.4 cm) for the non-nTMS group (*p* < 0.0001; Figure 2A). Lateral craniotomy extension was 3.4 ± 1.3 cm (median 4.0 cm, range 1.2–6.5 cm) for the nTMS and 4.0 ± 1.9 cm (median 3.9 cm, range 1.3–11.0 cm) for the non-nTMS group (*p* = 0.0166; Figure 2B). Overall size of the bone flap was 16.7 ± 8.6 cm^2^ (median 16.0 cm^2^, range 5.1–52.5 cm^2^) for the nTMS and 25.0 ± 17.1 cm^2^ (median 20.0 cm^2^, range 6.7–92.0 cm^2^) for the non-nTMS group (*p* < 0.0001; Figure 2C).

#### Duration of Surgery

Duration of surgery from incision to end of suture was 128.8 ± 49.4 min (median 128.0 min, range 30.0–255.0 min) for nTMS and 158.0 ± 65.8 min (median 143.0 min, range 53.0–518.0 min) for non-nTMS patients (*p* = 0.0002).

### Overall Clinical Outcome in Motor Function

**Figure 3:**
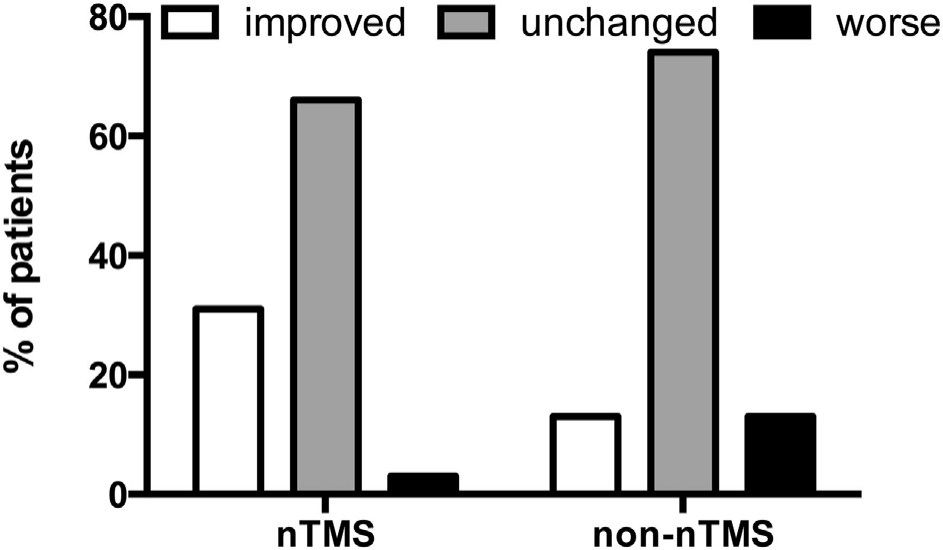
**Overall outcome for motor function**. This bar chart demonstrates the percentage of patients in which paresis in the navigated transcranial magnetic stimulation (nTMS) and non-nTMS group improved, remained unchanged, or worsened compared to the preoperative state (*p* = 0.0002).

In the nTMS group, there were three patients (2.5%) who suffered from a new surgery-related transient paresis and five patients (3.8%) in the non-nTMS group. Concerning new permanent pareses, there were 4 patients (3.3%) in the nTMS and 17 patients (13.1%) in the non-nTMS group (*p* = 0.0160). We also performed a sub-analysis of improvement of preoperative pareses. In the nTMS group, 37 patients (30.8%) improved and 79 (65.8%) remained unchanged; in the non-nTMS group, 17 patients (13.1%) improved, while 96 patients (73.8%) remained unchanged. Moreover, on long-term follow-up of all patients, 4 patients (3.4%) in the nTMS group and 17 patients (13.1%) in the non-nTMS group demonstrated a worsened paresis (*p* = 0.0002; Table 2; Figure 3).

**Table 2:** **Postoperative course**.

		nTMS	Non-nTMS	*p*-Value
Residual tumor (%)		7.7	21.6	0.0024
Unexpected residual (%)		3.4	18.4	0.0002
Surgery-related paresis (%)	Improved	30.8	13.1	0.0002
	Unchanged	65.8	73.8	
	Worse	3.4	13.1	
	None	94.2	83.1	0.0160
	Transient	2.5	3.8	
	Permanent	3.3	13.1	
Surgery-related complications on magnetic resonance imaging (%)	None	94.2	68.0	<0.0001
	Hemorrhage	0.0	13.7	
	Infection	3.3	5.2	
	Revision surgery	2.5	13.1	
GTR	New permanent paresis (%)	3.7	11.2	0.0380
	No new permanent paresis (%)	96.3	88.8	
STR	New permanent paresis (%)	0.0	22.2	0.1213
	No new permanent paresis (%)	100.0	77.8	

*The postoperative course of both groups, navigated transcranial magnetic stimulation (nTMS) compared to the non-nTMS group, is given. Residual tumor, unexpected residual, surgery-related paresis, and surgery-related complications as shown by postoperative imaging are displayed. Reasons for revision surgery were hemorrhage and corticospinal fluid leakage. In addition, the percentage of patients suffering from new permanent motor deficit after subtotal (STR) or gross total resection (GTR) according to postoperative imaging for both the nTMS and non-nTMS group is displayed*.

### Preoperative Paresis vs. Permanent Surgery-Related Deficit

**Figure 4:**
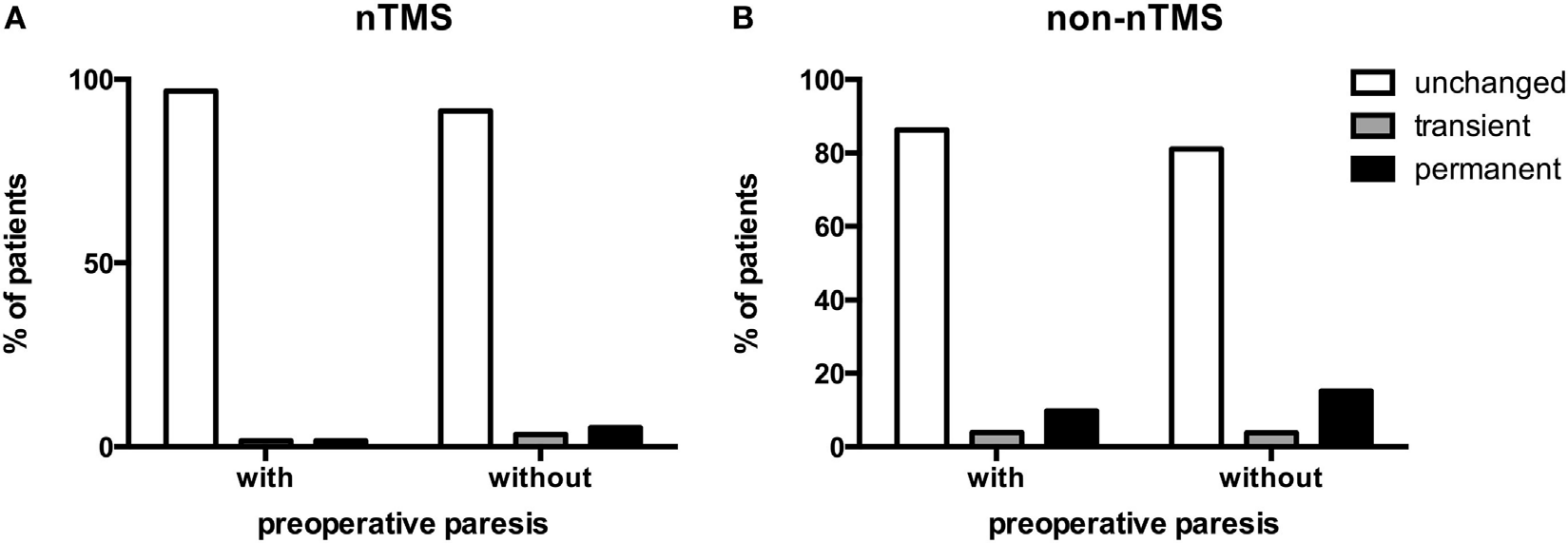
**Preoperative paresis vs. permanent surgery-related deficit**. This bar chart shows the percentage of patients with and without a preoperative paresis in the navigated transcranial magnetic stimulation (nTMS) **(A)** and the non-nTMS group **(B)**. On long-term follow-up, these pareses can be unchanged, transient, or permanent in comparison to the preoperative neurological status.

Preoperative pareses were comparable in both groups (Table 1). However, we examined whether clinical outcome might differ in both groups depending on preoperative pareses. Concerning the effect on functional outcome by the rate of new surgery-related pareses, we found 62 patients (51.7%) with preoperative paresis in the nTMS and 51 patients (39.2%) in the non-nTMS group (Table 1). On long-term follow-up, one patient (1.6%) in the nTMS group with preoperative paresis deteriorated permanently, compared to three patients (5.2%) without preoperative paresis. One patient (1.6%) with paresis and 2 patients (3.4%) without paresis showed a transient surgery-related paresis, while 60 patients (96.8%) with and 53 patients (91.4%) without preoperative paresis showed no surgery-related change in motor function on long-term follow-up (*p* = 0.4414; Figure 4A).

With regard to the non-nTMS group, there was no significant correlation between preoperative and new surgery-related paresis. Five patients (9.8%) in the non-nTMS group with preoperative paresis deteriorated permanently, compared to 12 patients (15.2%) without preoperative paresis. Two patients (3.9%) with paresis and 3 patients (3.8%) without paresis showed a transient surgery-related paresis, while 44 patients (86.3%) with and 64 patients (81.0%) without preoperative paresis showed no surgery-related change in motor function on long-term follow-up (*p* = 0.6730; Figure 4B).

### Tumor Location vs. Permanent Surgery-Related Paresis

The location of the metastasis was not associated with a higher rate of permanent new paresis in the nTMS (*p* = 0.2442) or non-nTMS group (*p* = 0.6615).

### Permanent New Surgery-Related Deficit vs. EOR

We observed a significantly lower rate of residual tumor in the nTMS group (Table 2). There were 9 patients (7.7%) in the nTMS and 27 patients (21.6%) in the non-nTMS group with residual tumor in postoperative scans (OR 0.3025; CI 0.1356–0.6749). Concerning expected EOR, there was no difference between groups: for 111 patients (95.7%) in the nTMS and 124 patients (96.1%) in the non-nTMS group, the surgeon expected GTR (OR 0.8952; CI 0.2524–3.175). However, there were 4 patients (3.4%) in the nTMS and 23 patients (18.4%) in the non-nTMS group with unexpected residual tumor on postoperative MRI (OR 0.1570; CI 0.0525–0.4694; Table 2). Concerning EOR and the relation to any new surgery-related permanent paresis, there was no significant difference between the nTMS (OR 0.8182; CI 0.04086–16.38) and the non-nTMS group (OR 0.4425; CI 0.1468–1.334) between GTR and subtotal resection (STR). However, when comparing new surgery-related permanent paresis for the nTMS and non-nTMS group separately after GTR and STR, we observed a significantly lower rate of new surgery-related permanent paresis in the nTMS group (OR 0.3042; CI 0.0935–0.9895). This difference was not found in the subgroup of patients who underwent STR (OR 0.1741; CI 0.0089–3.416; Table 2).

### Intraoperative Use of Neuromonitoring

Since the data were partially analyzed retrospectively, we were not able to assess in all cases whether IOM was used or not. Thus, in terms of IOM, we only differentiate between “yes” and “no/data missing.” By doing so, we observed a significantly lower rate of IOM in the nTMS group. There were 59 patients (49.2%) in the nTMS and 83 patients (62.9%) in the non-nTMS group in which IOM was used, while there was no IOM or data missing in 61 patients (50.8%) in the nTMS and 49 patients (37.1%) in the non-nTMS group (OR 0.571; CI 0.3453–0.9441).

## Discussion

The broad spectrum of pathologies within neurooncology presents a particular challenge when validating a new technique such as nTMS. Variations in tumor location, aggressiveness, and prognosis mean that a novel presurgical technique must undergo extensive testing in a large cohort before it can be considered effective. nTMS has demonstrated benefit in the surgical treatment of eloquent supratentorial primary central nervous system (CNS) lesions (2–4). It is as yet unclear whether similar benefit exists for the management of eloquent supratentorial metastatic lesions (2–4).

In the recent past, surgical resection of metastatic lesions in the CNS was undertaken routinely without any sort of IOM. Subsequently, studies showed the infiltrative character of brain metastases and the value of IOM (19–22). However, convincing data on the influence of preoperative mapping on clinical outcome were not available.

### Surgical Time

The difference in surgical time that we observed in this study may be a result of smaller craniotomy size and a reduced rate of intraoperative mapping and monitoring via DCS. Having nTMS motor mapping data available, we found that intraoperative mapping took on more of a confirmatory than an investigative role. Similarly, having preoperative nTMS data allowed for limited craniotomy size because larger exposures simply for mapping purposes were not required (6). Prior comparisons between large cohorts of patients undergoing nTMS and non-nTMS (for various primary CNS pathologies including eloquent gliomas) were not able to show any significant difference in surgical time (2–4). We postulate that this difference is due to the infiltrative nature of gliomas—in these cases, surgeons depend on IOM to give the most complete resection of abnormal tissue within eloquent cortex. Conversely, in resections of metastatic lesions, the border between tumor and non-tumor is usually defined, so mapping is most useful only while approaching and accessing the tumor. Thus, the surgeon may depend more upon a preoperative nTMS map so long as it is confirmed intraoperatively with IOM.

### Intraoperative Neuromonitoring

Although this data set is incomplete, the results reflect a clinical experience that became commonplace at each of the participating institutions over the course of the study. Namely, when preoperative nTMS maps clearly demonstrate the functional anatomy, they can then be swiftly confirmed after craniotomy (before tumor resection) and extensive motor mapping (or in some cases even motor monitoring) becomes superfluous (Figure 1). This experience is also reflected in the shorter surgical time of the nTMS group. However, it must be emphasized that nTMS is not meant to replace DCS mapping nor motor monitoring. Instead, our findings show that nTMS can help to identify patients in which DCS mapping or motor monitoring might be unnecessary because of the distance between lesion and functionally eloquent motor regions. Similarly, if the lesion is in close proximity to eloquent motor regions, nTMS can make intraoperative DCS more targeted and therefore quicker. Both these situations result in shorter surgical times and a higher level of certainty regarding the location of functional tissue in the nTMS cohort. It must also be mentioned that, in some cases, nTMS identifies a previously unrecognized motor region, thus leading to DCS mapping or motor monitoring where it might not have been otherwise employed. Thus, nTMS should be regarded as a supplementary tool, which complements IOM and improves patient safety.

### Craniotomy

Since extensive intraoperative mapping is usually not required when nTMS data are available, the surgeon can limit the craniotomy to the tumor instead of additionally exposing the precentral gyrus. This effect on craniotomy size with respect to the exposure of primary motor cortex is reflected in the smaller AP craniotomy extension compared to the lateral craniotomy extension when comparing the nTMS to the non-nTMS group (Figure 2).

### Tumor Residual

The EOR that we observed in this study is in accordance with other studies on the effect of mapping on EOR (2–4, 23–25). Studies have shown conclusively that IOM results in greater EOR (6, 23, 26); how the addition of preoperative mapping further improves EOR is not as obvious. Most likely, the concordance of a preoperative, nTMS-based map with an intraoperative DCS map offers greater confidence in the cortical and subcortical anatomy, thereby leading to more extensive resection.

### New Surgery-Related Pareses

Average follow-up was comparable in both groups and sufficiently long to exclude further recovery of motor function. Thus, the observed long-term pareses can be judged as permanent (Table 2).

By showing that preoperative mapping reduces the risk of surgery-related deficits, this study correlates well with other available studies by Duffau, De Witt Hamer, and others proving that additional functional mapping reduces surgery-related pareses in patients with motor eloquent lesions (2, 4, 23–25). The rates of postoperative neurological improvement in the nTMS group were more than twice that of the non-nTMS group (Figure 3; Table 2). The overall high improvement rate is well in accordance with other data on brain metastases (1, 19). However, significant difference in improvement between nTMS and non-nTMS patients bears additional discussion (Figure 3; Table 2). In general, reducing the mass effect and tumor-induced edema of brain metastases causes recovery of the motor system in many cases. Previous studies in glioma patients have shown that nTMS data, especially when combined with DTI FT, changes the surgical approach in a considerable number of cases because functional motor tracts were identified in unexpected locations. Thus, approach planning in the non-nTMS group can put at risk both the motor eloquent cortex and the CST. We hypothesize that this additional benefit of nTMS explains the differential rates of motor recovery between the two groups (Figure 3; Table 2).

### Alternative Mapping Techniques

nTMS is not the only non-invasive technique to map cortical motor function. Other techniques are MEG and fMRI. fMRI is a very broadly available and frequently used modality. Some studies showed a good correlation between fMRI and intraoperative DCS in identifying the primary motor cortex (6, 16, 27–29). However, in contrast to MEG and nTMS, fMRI does not gauge electrophysiological cortical function. Rather, it visualizes increased oxygen consumption, which is a proxy for metabolic demand, which is a proxy for neurological activation. Thus fMRI is a surrogate parameter, and it can be severely influenced by ischemia, tumor infiltration, or edema, which can interfere with measurement of cortical function (30). For this reason, current consensus is that fMRI lacks the sensitivity and specificity to guide surgical resection in cortical areas adjacent to intracerebral lesions, and fMRI alone is not used for surgical planning (31–34).

In contrast, MEG has been shown to correlate well with both DCS and nTMS in mapping motor eloquent cortex (35). However, due to the high cost of MEG facilities, the availability of this modality to the majority of brain tumor patients is limited. nTMS, on the other hand, is comparably economical and easy to use. Thus, its widespread availability opens new options for non-invasive mapping. Because it relies on MEPs, it is a true test of function rather than a proxy for function. As such, its methodology is comparable to DCS, the gold standard in the neurosurgical community (36–40).

### Limitations of Our Data

Since nTMS and the intraoperative use of the functional data requires neuronavigation, the precision of intraoperatively displayed motor mapping data can be impaired by several confounding factors. First of all, these data harbor the accumulated registration errors and navigation errors of the nTMS and the intraoperative neuronavigation system. Moreover, intraoperative neuronavigation usually shows brain shift beginning at some point after durotomy (41, 42). However, since brain shift occurs some time after durotomy, it does not weaken the practical use of nTMS data. The nTMS data are useful primarily for assessing the spatial relationship between tumor and CST, for craniotomy planning, and for identifying the primary motor cortex right after durotomy. Subsequent to its identification, the primary motor cortex can be recognized visually for the remainder of tumor resection. Therefore, brain shift does not interfere with the major applications of nTMS data.

Another limitation of our results is that the intraoperative use of IOM could not be evaluated completely. For example, data regarding the type of IOM, the frequency of stimulation, the extent of mapping, etc., were not available for analysis. It should be noted, however, that the main focus of this study was not to compare techniques of IOM, but rather to delineate the impact of adding preoperative nTMS to the current standard of care. Despite the observed lower rate of IOM in the nTMS group, the influence of nTMS data on IOM has to be evaluated in a separate study in order to obtain more reliable data since this issue was not the main focus of the study reported in this manuscript.

This study is also subjected to the limits of a prospective, non-randomized observational cohort study. In order to demonstrate conclusively the value of nTMS, a randomized controlled trial comparing patients with or without nTMS is required. It should be noted, however, that neither DCS nor intraoperative neuronavigation have level I evidence yet both are considered standard of care seen as mandatory by most neurosurgeons. For intraoperative mapping by DCS the best existing level of evidence is also a matched pair analysis with a historic control group (23). Thus, while a randomized controlled trial as discussed earlier would be ideal, we encourage clinicians not to wait for such a trial to adopt nTMS into routine clinical practice.

Nonetheless, a randomized trial would also help us to look further into important questions raised by the data of this study, such as “What is the value of nTMS in decision making to offer surgery versus radiosurgery or radiation therapy alone to lesions in eloquent cortex?” or “How many patients were considered for surgical resection and aborted due to results of nTMS?”

### Perspective of nTMS in Neurosurgery

The use of nTMS as a preoperative mapping tool in neurosurgical patients has gained attention in the last 5 years. During this time, the benefits of having precise preoperative functional data—especially for motor function—have gained widespread recognition. These maps offer an unprecedented ability to identify functional motor pathways and visualize the spatial relationships between tumor and adjacent eloquent structures. This ability has implications not only for preoperative and intraoperative planning but also for the discussion of perioperative risk. Thus, the value of nTMS data cannot be measured by outcome data alone; it also allows for better patient preparation. Furthermore, it fosters a frank discussion between patient and surgeon regarding the risk–benefit ratio of radical tumor resection vs. preservation of function.

Studies have demonstrated that nTMS improves functional and oncological outcome for patients with motor eloquent primary CNS lesions (2–4). This study demonstrates similar benefit within the subgroup of patients with metastases. From an oncological perspective, nTMS opens up the potential for tracking tumor-induced plasticity and reorganization of the motor system. The occurrence of such functional reorganization has been described using IOM; gaining this information non-invasively, however, would allow a clinician to identify the earliest time point after an initial STR when a repeat resection might confer maximal benefit with minimal risk (5, 43–47).

## Conclusion

This non-randomized study suggests that preoperative motor mapping by nTMS may improve the treatment of patients undergoing surgical resection of metastases in peri-Rolandic regions. These findings suggest that further evaluation with a prospective, randomized trial may be warranted. Further study would also allow for quantification of the economic impacts of shorter surgical time, a reduced need for intraoperative DCS mapping, and improved patient experience during the perioperative period.

## Author Contributions

SK, TP, and PT drafted the manuscript and its final revision and are also responsible for concept and design. All the authors were responsible for data acquisition, performed data analysis and clinical assessment, and approved and corrected the final version of the manuscript.

## Conflict of Interest Statement

SK, TP, and FR are consultants for BrainLAB AG (Feldkirchen, Germany). SK and TP are consultants for Nexstim Oy (Helsinki, Finland). The authors report no conflict of interest concerning the materials or methods used in this study or the findings specified in this manuscript.

## Abbreviations

AP, anterior–posterior; BMRC, British Medical Research Council; CI, confidence interval; CNS, central nervous system; CST, corticospinal tract; CT, computed tomography; DCS, direct cortical stimulation; DTI, diffusion tensor imaging; DTI FT, diffusion tensor imaging fiber tracking; EMG, electromyography; EOR, extent of resection; GTR, gross total resection; fMRI, functional magnetic resonance imaging; IOM, intraoperative neuromonitoring; MEG, magnetoencephalography; MEP, motor-evoked potential; MRI, magnetic resonance imaging; nTMS, navigated transcranial magnetic stimulation; OR, odds ratio; rMT, resting motor threshold; SRS, stereotactic radiosurgery; STR, subtotal resection; TMS, transcranial magnetic stimulation.
